# Deep Learning-Based Dental Caries Diagnosis on Panoramic Radiographies: Performance of YOLOv8 Versus Human Observers

**DOI:** 10.3390/diagnostics16081150

**Published:** 2026-04-13

**Authors:** Kader Biçengil, Ayça Kurt, Muhammed Enes Naralan, İrem Okumuş

**Affiliations:** 1 Hospitadent Dental Hospital, Istanbul 34893, Türkiye; k.ozbozkurt13@gmail.com; 2 Department of Pedodontics, Faculty of Dentistry, Recep Tayyip Erdogan University, Rize 53020, Türkiye; irem.okumus@erdogan.edu.tr; 3 Department of Oral and Dentomaxillofacial Radiology, Faculty of Dentistry, Recep Tayyip Erdogan University, Rize 53020, Türkiye; muhammedenes.naralan@erdogan.edu.tr

**Keywords:** artificial intelligence, deep learning, dental caries, diagnosis, panoramic radiographies

## Abstract

**Objectives**: To evaluate the diagnostic performance of a YOLOv8x-based deep learning model for detecting approximal, occlusal and buccal caries on paediatric panoramic radiographs and to compare its performance with human observers with different levels of clinical experience. **Methods**: A total of 1526 panoramic radiographs obtained from children aged 5–12 years were retrospectively analysed. Approximal, occlusal, and buccal caries in primary molars were annotated and used to train a YOLOv8x object-detection model. Model performance was evaluated on an independent test set and compared with three human observers: an intern dentist (ID), a novice specialist student (NSS), and an experienced specialist student (ESS). Diagnostic performance was assessed using precision, sensitivity, F1 score, and true positive counts. **Results**: The YOLOv8x model demonstrated moderate performance in detecting approximal caries (F1 score: 0.576) but showed limited performance for occlusal caries (F1 score: 0.24) and failed to detect buccal caries. The AI model showed lesion-dependent performance. For approximal caries, it performed comparably to ESS observers (*p* > 0.05) and better than ID (*p* < 0.001). Performance was poor for buccal caries (*p* < 0.001), and intermediate for occlusal caries, with no difference from NSS or ESS (*p* > 0.05) but lower than ID (*p* < 0.001). Overall, performance was comparable to experienced observers (*p* > 0.05) and superior to less experienced observers (*p* < 0.001). **Conclusions**: The YOLOv8x model achieved diagnostic performance comparable to less experienced clinicians in detecting dental caries on paediatric panoramic radiographs but did not reach expert-level accuracy. These findings suggest that deep learning models may serve as supportive tools in panoramic caries assessment rather than replacements for expert interpretation.

## 1. Introduction

Dental caries remains one of the most prevalent chronic diseases in children worldwide and continues to pose a significant diagnostic challenge, particularly in the mixed and primary dentition [1,2,3]. Dental caries is a multifactorial, infectious disease that has negative effects on the general health, development and quality of life of children who continue to grow and develop [4,5,6]. Accurate radiographic detection of carious lesions is essential for early diagnosis and appropriate treatment planning, yet radiographic interpretation is influenced by anatomical factors, image quality, and observer experience [7]. However, its diagnostic accuracy for detecting dental caries—especially approximal, occlusal, and buccal lesions—is limited when compared with bitewing or periapical radiographs [3,8]. Superimposition, lower spatial resolution, and anatomical variations in primary teeth further complicate reliable caries detection on panoramic images [9,10].

Previous studies have demonstrated considerable variability in caries detection accuracy among dental students, general dentists, and specialists, indicating a strong dependence on clinical experience [11,12,13]. The examination of X-ray images in clinical practice increases the workload of dentists [14]. Clinical workload, time constraints, and increasing patient numbers are other significant factors that make radiographic evaluation challenging [15]. Considering all of this, it can be said that the accuracy of radiological diagnosis is influenced by various factors.

In recent years, deep learning-based artificial intelligence (AI) systems have emerged as promising tools for automated image analysis in dentomaxillofacial radiology [14,16]. Convolutional neural networks (CNNs) have shown encouraging results in detecting caries, periapical lesions, and periodontal bone loss on intraoral and panoramic radiographs [17,18]. The development of automated systems capable of objectively diagnosing caries with excellent consistency and accuracy is crucial for healthcare applications, given the benefits it would provide. Several studies have reported high performance of AI models in detecting approximal caries [12,13,19]; however, evidence regarding automated detection of occlusal and buccal caries remains limited [14,20,21]. Moreover, most existing studies have focused either on a single caries type or on comparisons with experienced clinicians, while limited attention has been given to comparing AI performance across observers with varying levels of clinical experience [11,12,13]. Evaluating AI systems against human observers at different training stages is essential to understand their potential role in routine clinical workflows.

Therefore, the aim of this study is to compare the performance of an AI-trained YOLOv8x model in detecting buccal, occlusal, and interproximal caries with the diagnostic performance of pediatric dentistry researchers and student dentists of varying experience levels in the same caries types. The null hypothesis was that there would be no significant difference in diagnostic performance between the YOLOv8x model and human observers with varying clinical experience. The alternative hypothesis was that significant differences would exist between AI-based and human caries detection performance.

## 2. Materials and Methods

### 2.1. Study Design and Ethical Approval

This retrospective diagnostic accuracy study was approved by the Recep Tayyip Erdoğan University Non-Invasive Clinical Research Ethics Committee (Decision No: 23.05.2024/111). The study protocol complied with the Declaration of Helsinki (1975, revised 2013). Institutional permission was obtained to use archived panoramic radiographic data. Written informed consent for diagnostic imaging had been obtained from the parents or legal guardians of all paediatric patients at the time of image acquisition.

### 2.2. Study Population and Image Acquisition

Panoramic radiographs of paediatric patients aged 5–12 years, acquired between 2018 and 2024 for routine diagnostic purposes, were retrospectively reviewed. All images were obtained using the same panoramic imaging unit (Planmeca ProMax 2D S2, Planmeca Oy, Helsinki, Finland) with standardized exposure parameters (66 kVp, 8 mA, 16.6 s).

Radiographs were included if they met the following criteria:

1. Patient age between 5 and 12 years;
2. Absence of motion artefacts or severe image distortion;
3. Presence of at least one primary molar in any quadrant;
4. Absence of radiographic findings suggestive of syndromic conditions (e.g., ectodermal dysplasia, cleidocranial dysostosis, Down syndrome).

Teeth with extensive coronal destruction, presenting as residual roots, were not included in the annotation process because the original crown morphology could not be reliably evaluated on panoramic radiographs. Radiographs that did not meet these criteria were excluded from the study by an Oral and Dentomaxillofacial Radiologist (M.E.N.) with 5 years of professional experience. Images were not stratified according to sex or ethnicity.

### 2.3. Dataset Composition and Splitting

A total of 1533 panoramic radiographs meeting the inclusion criteria were initially identified. Seven radiographs without any detectable carious lesions in primary molars were excluded from model training to prevent class imbalance bias. Seven panoramic radiographs of primary molars without caries were excluded from the training dataset to reduce class imbalance during model training. However, these images were retained in the overall dataset and were included in the annotation and evaluation processes. Consequently, 1526 panoramic radiographs were included for deep learning model development and evaluation.

To avoid data leakage, dataset splitting was performed on a patient basis, ensuring that images from the same patient did not appear in more than one subset. The dataset was randomly divided into training (80%), validation (10%), and test (10%) sets, resulting in 1222 images for training, 152 images for validation, and 152 images for independent testing.

### 2.4. Image Preprocessing

All panoramic radiographs were anonymised and stored in JPEG format. To ensure consistency in spatial resolution and feature extraction, all images were resized to a width of 1696 pixels while preserving aspect ratio. This resolution was selected to balance anatomical detail preservation with computational efficiency. No additional normalization or standardization was applied beyond resizing. Specifically, no contrast enhancement, filtering, histogram equalization, denoising, sharpening, or intensity standardization was performed prior to annotation or model training, in order to preserve routine clinical imaging conditions.

### 2.5. Caries Annotation and Ground Truth Definition

Approximal, occlusal and buccal caries lesions in primary molars were annotated using CranioCatch software (YOLOv8x-based, Eskişehir, Türkiye). In the first phase, three observers with different levels of clinical experience independently annotated the full dataset:

1. A final-year undergraduate dental student (Intern Dentist, ID);
2. A first-year paediatric dentistry specialist student (Novice Specialist Student, NSS);
3. A second-year paediatric dentistry specialist student (Experienced Specialist Student, ESS).

These annotations were used for training the deep learning model to reflect real-world interobserver variability in caries detection. These annotations were used for training the deep learning model to reflect real-world interobserver variability in caries detection. In the training phase, annotations generated independently by the three observers were retained as separate lesion labels rather than being merged into a single consensus annotation set. This approach was preferred in order to preserve interobserver variability and better reflect routine clinical interpretation.

For performance evaluation, a subset of 100 panoramic radiographs was randomly selected from the dataset. Ground truth annotations for this subset were established by a paediatric dentist with more than 10 years of clinical experience (A.K.). To strengthen annotation reliability, the images were also independently reviewed by an oral and maxillofacial radiologist with 5 years of experience (M.E.N.), and the final labels were determined by consensus between the two experts. To assess intra-observer reliability, A.K. re-annotated the same images after a two-week interval. Intra-observer agreement was evaluated using Cohen’s kappa coefficient (κ = 0.83), indicating almost perfect agreement.

### 2.6. Observer Performance Evaluation

All three observers (ID, NSS, ESS) independently re-annotated the 100-image test subset under identical viewing conditions. Observers were blinded to each other’s annotations and to the AI model outputs. Their diagnostic performance was compared with the expert-defined ground truth annotations.

### 2.7. Deep Learning Model Architecture and Training

A YOLOv8x object-detection architecture was employed for automated caries detection. YOLOv8x was selected due to its capability for single-stage detection, high computational efficiency, and suitability for identifying small objects within large-field images such as panoramic radiographs.

**Figure 1:**
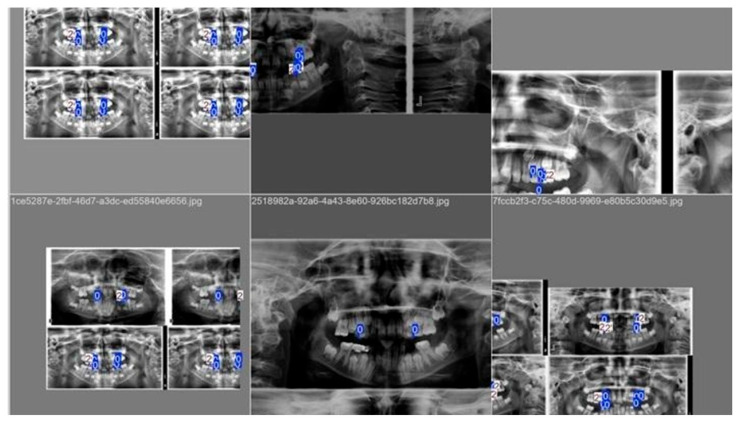
YOLOv8x AI model training phase snapshot.

The model was trained using 1526 panoramic images containing a total of 12,499 annotated caries labels (Figure 1). Training was performed using Python-based deep learning libraries, including PyTorch, OpenCV, NumPy, Pandas, TorchVision, and TensorBoard, implemented within the CranioCatch (YOLOv8x-based) platform.

**Figure 2:**
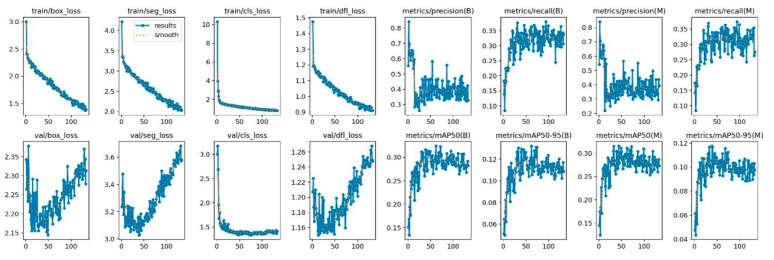
YOLOv8x caries detection performance graphs according to epoch progress.

Data augmentation techniques—HSV-Hue, Hue-Saturation, HSV-Value, and Mosaic augmentation—were applied during training to improve model robustness and generalisation. Training was configured for a maximum of 800 epochs; however, early stopping was implemented, and optimal performance was achieved at epoch 33, beyond which no further improvement was observed (Figure 2).

### 2.8. Model Evaluation and Performance Metrics

The independent test set (10% of the dataset) was used exclusively for performance evaluation. Model predictions were compared with expert-defined ground truth annotations to calculate true positives (TP), false positives (FP), and false negatives (FN).

Diagnostic performance was assessed using precision, sensitivity (recall), F1 score and mean average precision (mAP). Intersection over Union (IoU) was used to determine correct detections, with an IoU threshold of ≥0.50 adopted in accordance with established object-detection standards [22].

### 2.9. Additional Performance Analysis

Model performance was further evaluated using confusion matrices, bounding box visualisation, and mask-based segmentation analysis to assess localisation accuracy and lesion boundary delineation. Both object detection (bounding box) and segmentation (mask) outputs were analysed to explore the suitability of YOLOv8x for panoramic caries assessment [23,24].

### 2.10. Statistical Analysis

All statistical analyses were performed in Python using the pandas, numpy, scikit-learn, and statsmodels libraries. To compare the AI model with each human observer, paired exact McNemar tests were performed. Two complementary McNemar analyses were conducted. First, prediction disagreement was assessed by comparing the binary decisions of the AI model and each human observer, regardless of reference correctness. Second, diagnostic correctness was compared relative to the reference standard by classifying each paired decision as correct or incorrect; this analysis was considered the primary inferential comparison for determining whether the diagnostic correctness of the AI model differed from that of the human observers. To control for multiple testing, *p*-values obtained from the McNemar and paired bootstrap comparisons were adjusted using the Benjamini–Hochberg procedure. A two-sided adjusted *p*-value of less than 0.05 was considered statistically significant.

## 3. Results

### 3.1. Approximal Caries Detection

For approximal caries, TP counts were 226 (ID), 275 (NSS), 415 (ESS), and 408 (AI) (Table 1). ESS showed the lowest false positives and the highest precision, sensitivity, and F1 score (0.709), while the AI model had an F1 score of 0.576. As shown in Table 2, diagnostic performance varied considerably according to lesion type and evaluator. For approximal caries, the AI model achieved the highest sensitivity (0.860) and the highest F1 score (0.889), with precision (0.919) comparable to that of the human observers. The pairwise exact McNemar test based on diagnostic correctness (Table 3) showed that the comparative performance of the AI model depended on lesion type. For approximal caries, no significant difference was found between the AI model and NSS or ESS (*p* = 0.706 and 1.000, respectively), whereas the AI model performed significantly differently from ID (*p* < 0.001) (Table 3). Paired bootstrap comparisons of recall and F1 score (Table 4) further clarified the direction and magnitude of these differences. For approximal caries, the AI model demonstrated significantly higher recall than ESS and ID, and a significantly higher F1 score than ID; however, its F1 score did not differ significantly from NSS or ESS (Table 4).

### 3.2. Buccal Caries Detection

In buccal caries detection, the TP counts were 6 for ID, 2 for NSS, and 12 for ESS. (Table 1). The AI model showed very low sensitivity (0.022) despite perfect precision (1.000), resulting in the lowest F1 score (0.043) among all evaluators (Table 2). The AI model differed significantly from NSS and ID after multiple-testing correction (*p* < 0.001), but not from ESS (*p* = 0.706) (Table 3). The AI model showed significantly lower recall than all three human observers, with significant reductions in F1 score relative to NSS and ESS, whereas the F1 difference versus ID was not statistically significant after correction (Table 4).

### 3.3. Occlusal Caries Detection

For occlusal caries detection, TP counts were 53 for ID, 59 for NSS, 82 for ESS, and 69 for the AI model. The highest F1 score was observed for ESS (0.464), while the lowest F1 score was recorded for the AI model (0.240) (Table 1). The AI model showed lower sensitivity (0.424) but relatively high precision (0.820), yielding an intermediate F1 score (0.559) (Table 2). For occlusal caries, no statistically significant difference in correctness was observed between the AI model and NSS or ESS after adjustment (*p* = 0.087 and 0.222, respectively), while the comparison with ID remained significant (*p* < 0.001) (Table 3). The AI model had significantly lower recall than all three human observers, but its F1 score was significantly lower only than ESS, while the differences versus NSS and ID were not significant (Table 4).

### 3.4. Overall Diagnostic Performance

**Figure 3:**
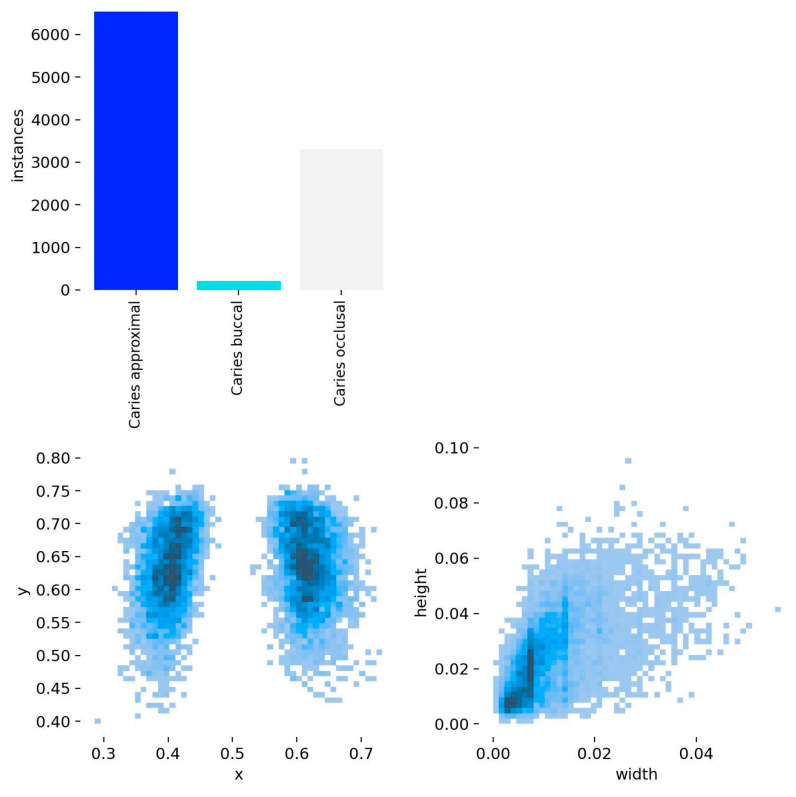
Distribution of caries lesion types in the dataset. The bar chart shows the frequency of caries types (approximal, buccal, and occlusal). The lower plots illustrate the spatial distribution (x–y coordinates) and size (width–height) of the detected regions. Color intensity represents data density, where darker areas indicate higher concentrations of observations and lighter areas indicate lower concentrations.

Overall TP counts were 509 for ESS, 477 for the AI model, 336 for NSS, and 285 for ID. Of all caries types, the lowest TP value was observed in buccal caries (ID:6, NSS:2, ESS:12, AI:0). Precision and sensitivity were highest in the ESS group and lowest in the ID group. The overall F1 scores were 0.639 for ESS, 0.378 for NSS, 0.329 for ID, and 0.472 for the AI model (Table 1). When lesion-level decisions were pooled across all lesion types, the AI model demonstrated an overall sensitivity of 0.712, precision of 0.905, F1 score of 0.797, and accuracy of 0.885, indicating overall performance close to that of ESS and superior to that of ID, although still slightly lower than ESS in pooled F1 score and accuracy (Table 2). In the overall pooled analysis across lesion types, the AI model differed significantly from NSS and ID (*p* < 0.001), but not from ESS (*p* = 0.407), indicating that the overall diagnostic correctness of the AI model was comparable to that of ESS, but not to that of the other human observers (Table 3). In the overall pooled analysis, the AI model showed slightly lower recall but a significantly higher F1 score than NSS, no significant difference from ESS in either recall or F1 score, and a significantly higher pooled F1 score than ID, whereas the recall difference versus ID was not significant (Table 4). Overall, these findings indicate that the AI model performed particularly well for approximal caries, poorly for buccal caries, and intermediately for occlusal caries, with pooled performance broadly comparable to that of ESS. Furthermore, the distribution of lesion types in the dataset was uneven; buccal caries represented a significantly smaller proportion of cases compared to proximal and occlusal lesions (Figure 3).

### 3.5. Inter-Observer Agreement and Disagreement Analysis

Supplementary Table S1 presents the pairwise exact McNemar comparisons based on prediction disagreement between the AI model and the human observers, irrespective of reference correctness. For buccal caries, the AI model differed significantly from all three human observers (*p* < 0.001), reflecting markedly different positive/negative decision patterns. For approximal caries, prediction disagreement was not significant between the AI model and NSS after multiple-testing correction (*p* = 0.098), whereas significant disagreement was observed between the AI model and both ESS and ID (*p* < 0.001). For occlusal caries, the AI model again differed significantly from all human observers (*p* < 0.001). In the overall pooled analysis across lesion types, significant prediction disagreement was found between the AI model and NSS as well as ID (*p* < 0.001), whereas no significant disagreement was observed between the AI model and ESS (*p* = 0.946). Overall, these findings suggest that the binary decision pattern of the AI model was most similar to that of ESS and least similar to that of ID (Supplementary Information—Table S1).

## 4. Discussion

This study evaluated the diagnostic performance of a YOLOv8x deep learning model for dental caries detection on panoramic radiographs and compared its performance with observers of different clinical experience levels. The findings demonstrate that caries detection accuracy varies substantially according to lesion type and observer experience, and that AI systems currently do not uniformly outperform experienced clinicians when panoramic imaging is used. Overall, the YOLOv8x model achieved higher F1 scores than ID and NSS but remained inferior to ESS. The superior performance demonstrated by ESS across most criteria reinforced the impact of clinical experience on radiographic diagnostic accuracy. Caries type significantly impacted diagnostic performance. Both human observers and the AI model showed the lowest accuracy for buccal caries. The moderate performance of the YOLOv8x model in approximal caries detection should be interpreted in light of the inherent limitations of paediatric panoramic radiography. Mixed dentition, anatomical superimposition, physiological root resorption, and developing permanent tooth germs may obscure early approximal lesions and reduce radiographic conspicuity [3,8,9,10]. This represents a relevant contribution, as routine paediatric panoramic radiographs remain underrepresented in AI-based caries research [12,13]. The findings suggest that AI-assisted approaches can contribute to clinical decision-making processes, especially given the challenges of detecting caries in panoramic images. The superior performance demonstrated by ESS across most criteria reinforced the impact of clinical experience on radiographic diagnostic accuracy. Caries type significantly impacted diagnostic performance. Both human observers and the AI model showed the lowest accuracy for buccal caries. In contrast, performance was markedly limited for buccal caries, where extremely low sensitivity resulted in poor detection despite high precision. For occlusal caries, the AI model demonstrated intermediate performance, characterized by relatively high precision but reduced recall compared to human observers. In the overall pooled analysis, the AI model achieved diagnostic performance comparable to that of ESS, while outperforming ID and NSS. Additionally, agreement analyses indicated that the decision-making pattern of the AI model was most similar to that of ESS and least similar to that of ID. These findings highlight both the potential of AI-assisted caries detection systems to reach expert-level performance and the importance of lesion characteristics and dataset distribution—particularly the underrepresentation of buccal caries—in shaping model performance.

Evaluating these findings within the framework of the literature is important for a better understanding of both the effect of observer experience on diagnostic accuracy and the comparative performance of AI-based systems with human observers. Our study makes a unique contribution to the literature by focusing on caries detection using panoramic radiographs, which are the most frequently used images in daily clinical practice, instead of idealized images, and by comparing the caries detection performance of dentists with various clinical experience levels using AI architecture. In pediatric dentistry, the presence of primary teeth and underlying permanent tooth germs in panoramic radiographs can lead to difficulties in diagnostic performance. Our literature review revealed that studies using AI to detect dental caries in children relied on photographic records and bitewing radiographs [25,26,27,28,29,30]. In this context, the present study, which utilizes panoramic radiographs of children and is performed with the YOLOv8x artificial intelligence architecture, is original and contributes to the limited number of studies [12,13,29] in the literature that have been conducted using panoramic radiographs of adults.

A systematic review evaluating the performance of AI algorithms in detecting dental caries reported an average sensitivity value of 66% [30]. One study reported higher accuracy rates of 94.59%, sensitivity of 72.26%, and specificity of 98.19% [29]. Another study in the literature reported different overall accuracy values of 80%, sensitivity of 75%, and specificity of 83% for interproximal caries [12]. In the current study, the average accuracy (62%) and sensitivity (38%) values were found to be lower compared to the results reported in previous studies. These differences may stem from methodological and clinical factors such as the type of image used, the characteristics of the dataset, the distribution of carious lesions, and especially the difficulties related to the anatomical structure of primary teeth.

In a study in the literature that examined interproximal caries in children and adults using bitewing radiographs with U-Net architecture, the average F1 score was reported as 0.543 and the average IoU score as 0.478 for adults, while the average F1 score was 0.440 and the average IoU score was 0.377 for children [26]. In the present study, the caries prediction performance of the YOLOv8x model, trained on panoramic radiographs of pediatric patients, was determined to be 0.576 (F1 score) and 0.541 (mAP@0.5 value), which is higher than the results reported in the literature for pediatric patient groups. This can be attributed to the superiority of the deep learning architecture used in object detection, training with high-resolution images, and the use of a large, labeled dataset. The performance difference between the studies may also stem from differences in the imaging method used (bitewing vs. panoramic), dataset size, labeling strategies, and evaluation metrics.

In the literature, AI-based caries detection in permanent teeth has been reported to have an overall accuracy of 94.59%, a sensitivity of 72.26%, and a specificity of 98.19% [29]. In a study including both pediatric and adult teeth, bitewing radiographs of primary and permanent teeth were labeled, and IoU values for primary teeth were reported as 25% for advanced caries, 17% for moderate caries, and 8% for incipient caries, while for permanent teeth, the values were reported as 51% for advanced caries, 19% for moderate caries, and 23% for incipient caries [26]. The study reported that AI architecture detected advanced caries more accurately in both primary and permanent teeth compared to other caries levels [26]. Another study in the literature reported that an AI model using R-CNN architecture had a sensitivity of %65 for enamel-level caries and %85 for advanced caries [13]. Studies have indicated that detection accuracy increases with increasing depth of carious lesions [13,26]. In studies where primary and permanent teeth are evaluated together, the reason why advanced caries are detected with higher accuracy in both primary and permanent teeth compared to incipient and intermediate lesions may be that the AI architecture is more successful in detecting significant radiographic changes. The relatively low sensitivity observed in this study may be partially attributed to the limited representation of advanced caries cases in the dataset, which may have affected the model’s ability to detect more severe lesions.

However, previous studies have also reported that the success of radiographic detection varies depending on the type of caries, and that diagnostic accuracy is lower on buccal and occlusal surfaces [31,32,33]. The literature contains a limited number of studies that use AI to detect buccal and occlusal caries [20,21]. A study using the DCDNet architecture for caries detection reported that AI model performed worse in detecting cervical caries [20]. In a study using the YOLOv8x deep learning architecture, a high sensitivity of 0.964 and an F1 score of 0.962 were reported in the detection of occlusal, cervical, and interproximal caries [21]. A review of the literature reveals that R-CNN and U-Net have been reported to exhibit high accuracy and specificity in detecting interproximal caries [12,13]. Another study using YOLOv3 reported high accuracy, sensitivity, and specificity in detecting interproximal caries [29]. YOLOv8x has also been reported to be successful in detecting interproximal caries [34,35]. The lower F1 scores obtained in the present study compared to some studies reported in the literature can primarily be explained by the differences in the type of radiographic image used. It is known that the vast majority of studies reporting high accuracy and F1 scores in the literature were conducted using bite-wing radiographs [19,20,21], which offer higher spatial resolution and less superposition, especially in the evaluation of interproximal caries [36]. In contrast, the use of panoramic dental radiographs obtained during routine clinical examinations in this study, while better reflecting actual clinical conditions, may have also presented diagnostic difficulties due to reduced contrast of carious lesions and increased anatomical superpositions. In current study, the lower performance observed in buccal and occlusal caries detection in the present study may be explained by both lesion-specific characteristics and imaging-related factors, particularly the use of panoramic radiographs, which are associated with lower contrast resolution and greater anatomical overlap compared to bite-wing images. This finding may be explained by several factors. First, the prevalence of buccal caries cases in the dataset was relatively low compared with other lesion types, which may have limited the model’s ability to learn representative features. Second, panoramic radiographs have inherent limitations in visualizing buccal surfaces due to superimposition and reduced radiographic contrast. These factors may have contributed to the poor performance of the model for this specific lesion type.

Studies conducted to measure the success of AI in detecting caries have reported differing opinions from observers [11,12,13,29]. One study reported accuracy values of 80% and 71% for CNNs and dentists, respectively [12]. The same study reported sensitivity values of 75% for CNN and 36% for dentists [12]. In a study comparing the R-CNN model with that of undergraduate students, it was found that the R-CNN achieved precision of 87%, sensitivity of 72%, and specificity of 93%, while the undergraduate students achieved precision of 82%, sensitivity of 47%, and specificity of 94% [13]. The literature has also investigated the effect of observer experience on diagnostic accuracy in radiographic caries diagnosis [11,37,38]. Studies have shown that the accuracy rate in detecting caries on radiography is directly proportional to the increase in professional experience [37,39,40]. Studies evaluating AI’s performance in detecting cavities have reported that while it cannot achieve the same level of accuracy as expert physicians, it shows higher accuracy than younger physicians [19,40]. However, the literature also reports that YZM shows superior success in caries detection compared to traditional methods [17]. In a study comparing general dentists and dental students in caries detection using the AI model ResNet-152, the highest accuracy 74% and specificity 92% were achieved by one of the dental student observers, while the second highest accuracy 72% and specificity 90% were reported for the AI model [11]. In contrast to a study in the literature that reported a higher specificity value for dentists than for R-CNN in interproximal caries detection [29], another study using U-Net reported that AI had higher sensitivity than dentists [12]. Another study using a deep learning model indicates that AI makes more accurate predictions than human observers [41]. Comparing the performance of AI-based systems with observers having varying levels of experience is becoming an increasingly important area of interest in the literature [11,29,39,41]. In the present study, the highest diagnostic accuracy was consistently achieved by experienced assistants, indicating that clinical expertise remains critical for interpreting panoramic radiographs.

A study in the literature comparing experienced dentists with AI reported that the AI model performed significantly better, with an average F1 score of 0.73 for AI and 0.41 for experienced dentists [12]. Similarly, it was reported that experienced dentists performed worse than AI, with an F1 score of 0.74 for R-CNN and 0.57 for undergraduate students in the study [13]. Our review of the literature revealed only one study comparing the caries detection performance of AI models in primary teeth by both experienced dentists and dental students [11]. In this study, where bitewing radiographs were labeled using the ResNet-152 architecture, the AI’s F1 score was reported as 0.55, which is higher on average than that of experienced dentists (0.37-0.50-0.58, respectively) and undergraduate students (0.50-0.53-0.58, respectively) [11]. In the present study, the YOLOv8x model, which exhibited a higher F1 score than the undergraduate student and the new assistant, did not achieve the caries detection success of the experienced assistant. Furthermore, in our study, the F1 scores of YOLOv8x were found to be lower than the F1 scores of AI models reported in the literature. In the present study, the use of panoramic radiographs may have led to blurring of the boundaries of carious lesions due to the dense anatomical superposition caused by primary teeth, physiological root resorption, and underlying permanent tooth germs. Although the YOLOv8x model outperformed less experienced observers, it did not reach expert-level performance, underscoring the current limitations of AI in complex imaging modalities.

Clinical implications, the findings suggest that deep learning models such as YOLOv8x may serve as supportive tools in panoramic radiograph interpretation, particularly for less experienced clinicians. AI-assisted systems could contribute to improved diagnostic consistency and reduce observer variability, especially for interproximal caries detection in pediatric patients.

This study has several limitations. First, all images were obtained from a single centre using a single panoramic unit with fixed acquisition parameters, which may limit the generalisability of the findings to other clinical settings, devices, and imaging protocols. Therefore, external validation using multicentre datasets acquired from different panoramic systems and imaging conditions is needed to better assess the robustness and clinical applicability of the proposed model. A second limitation is the exclusion of seven caries-free radiographs from the training set to reduce class imbalance, which may slightly affect the distribution of training samples. In addition, advanced caries (teeth with extensive coronal destruction and significant crown loss) were not labeled because lesion boundaries could not be reliably determined. While these decisions improved annotation reliability, they may limit comparability with studies including the full spectrum of caries severity and reduce real-world applicability. Moreover, using annotations from observers with different experience levels may introduce label variability and potential noise during training. Another limitation of anatomical superimposition inherent to pediatric panoramic imaging may have constrained both human and AI performance. Future studies incorporating multimodal imaging and lesion severity stratification are warranted.

## 5. Conclusions

These findings suggest that artificial intelligence systems may serve as supportive tools for clinicians, particularly in settings involving less experienced observers or high diagnostic workload. Rather than replacing expert interpretation, AI models currently appear more suitable as adjunctive tools that may improve diagnostic consistency in panoramic dental radiography. Future multicentre studies with larger, more balanced datasets and external validation are needed to improve clinical applicability.

## Figures and Tables

**Table 1:** Performance evaluation metrics of the AI model and human observers for each caries class.

Caries Class	PEM	ID	NSS	ESS	AI Model
Approximal Caries	TP	226	275	415	408
	FP	268	331	116	215
	FN	414	365	225	385
	Precision	0.458	0.454	0.782	0.655
	Sensitivity	0.353	0.430	0.648	0.515
	F1 Score	0.399	0.441	0.709	0.576
Buccal Caries	TP	6	2	12	0
	FP	133	60	11	7
	FN	40	44	34	22
	Precision	0.043	0.032	0.522	0
	Sensitivity	0.130	0.044	0.261	0
	F1 Score	0.065	0.037	0.348	0
Occlusal Caries	TP	53	59	82	69
	FP	149	152	61	72
	FN	157	151	128	365
	Precision	0.262	0.280	0.573	0.489
	Sensitivity	0.252	0.281	0.391	0.159
	F1 Score	0.257	0.280	0.465	0.240
Overall Results	TP	285	336	509	477
	FP	550	543	188	294
	FN	611	560	387	772
	Precision	0.341	0.382	0.730	0.619
	Sensitivity (Recall)	0.318	0.375	0.568	0.382
	F1 Score	0.329	0.379	0.639	0.473

PEM: Performance Evaluation Metrics; TP: True Positive; FP: False Positive; FN: False Negative; ID: Intern Dentist; NSS: Novice Specialist Student; ESS: Experienced Specialist Student; AI: Artificial intelligence.

**Table 2:** Diagnostic performance of the AI model and human observers by lesion type, including overall pooled performance across lesion types.

**Lesion Type**	**Reader**	***n***	**Sensitivity (95% CI)**	**Precision (95% CI)**	**F1 Score (95% CI)**	**Accuracy**
Buccal caries	NSS	800	0.222 (0.112–0.371)	0.161 (0.080–0.277)	0.187 (0.088–0.286)	0.891
Buccal caries	ESS	800	0.267 (0.146–0.419)	0.600 (0.361–0.809)	0.369 (0.207–0.511)	0.949
Buccal caries	ID	800	0.267 (0.146–0.419)	0.088 (0.046–0.148)	0.132 (0.064–0.201)	0.802
Buccal caries	AI	800	0.022 (0.001–0.118)	1.000 (0.025–1.000)	0.043 (0.000–0.140)	0.945
Approximal caries	NSS	800	0.835 (0.801–0.865)	0.930 (0.904–0.951)	0.880 (0.858–0.901)	0.845
Approximal caries	ESS	800	0.811 (0.775–0.843)	0.969 (0.949–0.983)	0.883 (0.861–0.904)	0.854
Approximal caries	ID	800	0.754 (0.715–0.789)	0.930 (0.902–0.952)	0.832 (0.806–0.856)	0.794
Approximal caries	AI	800	0.860 (0.828–0.888)	0.919 (0.892–0.942)	0.889 (0.868–0.909)	0.854
Occlusal caries	NSS	800	0.651 (0.575–0.722)	0.589 (0.516–0.660)	0.619 (0.558–0.677)	0.828
Occlusal caries	ESS	800	0.570 (0.492–0.645)	0.778 (0.695–0.847)	0.658 (0.594–0.716)	0.873
Occlusal caries	ID	800	0.587 (0.510–0.662)	0.510 (0.438–0.582)	0.546 (0.483–0.603)	0.790
Occlusal caries	AI	800	0.424 (0.350–0.502)	0.820 (0.725–0.894)	0.559 (0.482–0.627)	0.856
Overall pooled	NSS	2400	0.757 (0.725–0.787)	0.778 (0.747–0.808)	0.767 (0.744–0.791)	0.855
Overall pooled	ESS	2400	0.724 (0.691–0.756)	0.917 (0.892–0.938)	0.809 (0.785–0.831)	0.892
Overall pooled	ID	2400	0.687 (0.653–0.720)	0.674 (0.640–0.707)	0.681 (0.654–0.706)	0.795
Overall pooled	AI	2400	0.712 (0.679–0.744)	0.905 (0.878–0.927)	0.797 (0.773–0.820)	0.885

Overall pooled performance was calculated by pooling lesion-level decisions across buccal, approximal, and occlusal caries categories and recomputing the performance metrics from the aggregated counts. ID: Intern Dentist; NSS: Novice Specialist Student; ESS: Experienced Specialist Student; AI: Artificial intelligence.

**Table 3:** Pairwise comparison of diagnostic correctness between the AI model and human observers using the exact McNemar test.

**Lesion Type**	**Comparison**	***n***	**Discordant Pairs (AI Correct/Comparator Correct)**	**Exact McNemar *p***	**BH-Adjusted *p***
Buccal caries	AI vs. NSS	800	52/9	<0.001	<0.001
Buccal caries	AI vs. ESS	800	8/11	0.648	0.706
Buccal caries	AI vs. ID	800	125/11	<0.001	<0.001
Approximal caries	AI vs. NSS	800	73/66	0.611	0.706
Approximal caries	AI vs. ESS	800	63/63	1.000	1.000
Approximal caries	AI vs. ID	800	95/47	<0.001	<0.001
Occlusal caries	AI vs. NSS	800	75/52	0.050	0.087
Occlusal caries	AI vs. ESS	800	28/41	0.148	0.222
Occlusal caries	AI vs. ID	800	100/47	<0.001	<0.001
Overall pooled	AI vs. NSS	2400	200/127	<0.001	<0.001
Overall pooled	AI vs. ESS	2400	99/115	0.305	0.407
Overall pooled	AI vs. ID	2400	320/105	<0.001	<0.001

Discordant pairs indicate cases in which only one of the two readers compared was correct relative to the reference standard. BH-adjusted *p* values were calculated using the Benjamini–Hochberg procedure. ID: Intern Dentist; NSS: Novice Specialist Student; ESS: Experienced Specialist Student; AI: Artificial intelligence.

**Table 4:** Paired bootstrap comparison of recall and F1 score differences between the AI model and human observers.

**Lesion Type**	**Comparison**	**Metric**	***n***	**Difference (95% CI)**	**Bootstrap *p***	**BH-Adjusted *p***
Buccal caries	AI − NSS	Recall	800	−0.200 (−0.324 to −0.089)	<0.001	<0.001
Buccal caries	AI − NSS	F1 score	800	−0.143 (−0.254 to −0.035)	0.011	0.019
Buccal caries	AI − ESS	Recall	800	−0.244 (−0.373 to −0.125)	<0.001	<0.001
Buccal caries	AI − ESS	F1 score	800	−0.326 (−0.475 to −0.172)	<0.001	<0.001
Buccal caries	AI − ID	Recall	800	−0.244 (−0.378 to −0.119)	<0.001	<0.001
Buccal caries	AI − ID	F1 score	800	−0.088 (−0.176 to 0.010)	0.077	0.116
Approximal caries	AI − NSS	Recall	800	0.026 (−0.009 to 0.061)	0.166	0.209
Approximal caries	AI − NSS	F1 score	800	0.009 (−0.014 to 0.033)	0.442	0.506
Approximal caries	AI − ESS	Recall	800	0.050 (0.015 to 0.084)	0.006	0.010
Approximal caries	AI − ESS	F1 score	800	0.006 (−0.016 to 0.029)	0.602	0.629
Approximal caries	AI − ID	Recall	800	0.107 (0.071 to 0.140)	<0.001	<0.001
Approximal caries	AI − ID	F1 score	800	0.056 (0.032 to 0.081)	<0.001	<0.001
Occlusal caries	AI − NSS	Recall	800	−0.227 (−0.302 to −0.152)	<0.001	<0.001
Occlusal caries	AI − NSS	F1 score	800	−0.059 (−0.133 to 0.011)	0.104	0.146
Occlusal caries	AI − ESS	Recall	800	−0.145 (−0.219 to −0.074)	<0.001	<0.001
Occlusal caries	AI − ESS	F1 score	800	−0.098 (−0.167 to −0.033)	0.002	0.005
Occlusal caries	AI − ID	Recall	800	−0.163 (−0.246 to −0.081)	<0.001	<0.001
Occlusal caries	AI − ID	F1 score	800	0.013 (−0.064 to 0.087)	0.751	0.751
Overall pooled	AI − NSS	Recall	2400	−0.045 (−0.077 to −0.013)	0.004	0.008
Overall pooled	AI − NSS	F1 score	2400	0.030 (0.005 to 0.055)	0.022	0.035
Overall pooled	AI − ESS	Recall	2400	−0.012 (−0.043 to 0.020)	0.504	0.549
Overall pooled	AI − ESS	F1 score	2400	−0.012 (−0.035 to 0.011)	0.312	0.374
Overall pooled	AI − ID	Recall	2400	0.025 (−0.009 to 0.060)	0.150	0.199
Overall pooled	AI − ID	F1 score	2400	0.117 (0.090 to 0.144)	<0.001	<0.001

Differences were calculated as AI minus comparator. Positive values indicate higher performance for the AI model, whereas negative values indicate higher performance for the comparator. BH-adjusted *p* values were calculated using the Benjamini–Hochberg procedure. ID: Intern Dentist; NSS: Novice Specialist Student; ESS: Experienced Specialist Student; AI: Artificial intelligence.

## Data Availability

The original contributions presented in this study are included in the article/Supplementary Material. Further inquiries can be directed to the corresponding author.

## Supplementary Materials

The following supporting information can be downloaded at: https://www.mdpi.com/article/10.3390/diagnostics16081150/s1, Table S1: Pairwise comparison of prediction disagreement between the AI model and human observers using the exact McNemar test.

## Abbreviations

The following abbreviations are used in this manuscript:

CNN	Convolutional neural networks
AI	Artificial intelligence
ID	Intern dentist
ESS	Experienced specialist student
NSS	Novice specialist student
TP	True positives
FP	False positives
FN	False negatives

## References

[1] Broadbent J., Thomson W., Poulton R. (2008). Trajectory patterns of dental caries experience in the permanent dentition to the fourth decade of life. J. Dent. Res..

[2] American Academy of Pediatric Dentistry (AAPD) The State of Little Teeth in Report. https://www.mychildrensteeth.org/key-stats-state-of-little-teeth-report/.

[3] Selwitz R.H., Ismail A.I., Pitts N.B. (2007). Dental caries. Lancet.

[4] Fejerskov O., Nyvad B., Kidd E. (2015). Dental Caries: The Disease and Its Clinical Management.

[5] Petersen P.E., Bourgeois D., Ogawa H., Estupinan-Day S., Ndiaye C. (2005). The global burden of oral diseases and risks to oral health. Bull. World Health Organ..

[6] Filstrup S.L., Briskie D., da Fonseca M., Lawrence L., Wandera A., Inglehart M.R. (2003). Early childhood caries and quality of life: Child and parent perspectives. Pediatr. Dent..

[7] Beltrán-Aguilar E.D., Barker L.K., Canto M.T., Dye B.A., Gooch B.F., Griffin S.O., Hyman J., Jaramillo F., Kingman A., Nowjack-Raymer R. (2005). Surveillance for dental caries, dental sealants, tooth retention, edentulism, and enamel fluorosis—United States, 1988–1994 and 1999–2002. MMWR Surveill. Summ..

[8] Schwendicke F., Tzschoppe M., Paris S. (2015). Radiographic caries detection: A systematic review and meta-analysis. J. Dent..

[9] Stephens R. (1977). A comparison of panorex and intraoral surveys for routin dental radiography. J. Can. Dent. Assoc..

[10] Rushton V., Horner K., Worthington H. (1999). The quality of panoramic radiographs in a sample of general dental practices. Br. Dent. J..

[11] Nampim N., Panyarak W., Suttapak W., Wantanajittikul K., Nirunsittirat A., Wattanarat O. (2025). Deep learning-based caries lesion classification of primary teeth using bitewing radiographs and its comparison with dental professionals. Eur. Arch. Paediatr. Dent..

[12] Cantu A.G., Gehrung S., Krois J., Chaurasia A., Rossi J.G., Gaudin R., Elhennawy K., Schwendicke F. (2020). Detecting caries lesions of different radiographic extension on bitewings using deep learning. J. Dent..

[13] Chen X., Guo J., Ye J., Zhang M., Liang Y. (2022). Detection of Proximal Caries Lesions on Bitewing Radiographs Using Deep Learning Method. Caries Res..

[14] Oztekin F., Katar O., Sadak F., Yildirim M., Cakar H., Aydogan M., Ozpolat Z., Yildirim T.T., Yildirim O., Faust O. (2023). An explainable deep learning model to prediction dental caries using panoramic radiograph images. Diagnostics.

[15] Hintze H., Wenzel A. (2003). Diagnostic outcome of methods frequently used for caries validation: A comparison of clinical examination, radiography and histology following hemisectioning and serial tooth sectioning. Caries Res..

[16] Prados-Privado M., García Villalón J., Martínez-Martínez C.H., Ivorra C., Prados-Frutos J.C. (2020). Dental caries diagnosis and detection using neural networks: A systematic review. J. Clin. Med..

[17] Schwendicke F., Rossi J.G., Göstemeyer G., Elhennawy K., Cantu A., Gaudin R., Chaurasia A., Gehrung S., Krois J. (2021). Cost-effectiveness of Artificial Intelligence for Proximal Caries Detection. J. Dent. Res..

[18] Schwendicke F., Golla T., Dreher M., Krois J. (2019). Convolutional neural networks for dental image diagnostics: A scoping review. J. Dent..

[19] Mertens S., Krois J., Cantu A.G., Arsiwala L.T., Schwendicke F. (2021). Artificial intelligence for caries detection: Randomized trial. J. Dent..

[20] Dayı B., Üzen H., Çiçek İ.B., Duman Ş.B. (2023). A novel deep learning-based approach for segmentation of different type caries lesions on panoramic radiographs. Diagnostics.

[21] Karakuş R., Öziç M.Ü., Tassoker M. (2024). AI-assisted detection of interproximal, occlusal, and secondary caries on bite-wing radiographs: A single-shot deep learning approach. J. Imaging Inform. Med..

[22] Lee K., Kwak H., Oh J., Jha N., Kim Y., Kim W., Baik U., Ryu J. (2020). Automated detection of TMJ osteoarthritis based on artificial intelligence. J. Dent. Res..

[23] Redmon J., Divvala S., Girshick R., Farhadi A. You only look once: Unified, real-time object detection. Proceedings of the IEEE Conference on Computer Vision and Pattern Recognition.

[24] He K., Gkioxari G., Dollar P., Girshick R. (2020). Mask R-CNN. IEEE Trans. Pattern Anal. Mach. Intell..

[25] Lin H., Cui G., Liu Z., Pang L. (2024). Artificial intelligent model for detecting caries in primary teeth. Int. Dent. J..

[26] Azhari A.A., Helal N., Sabri L.M., Abduljawad A. (2023). Artificial intelligence (AI) in restorative dentistry: Performance of AI models designed for detection of interproximal carious lesions on primary and permanent dentition. Digit. Health.

[27] Moharrami M., Farmer J., Singhal S., Watson E., Glogauer M., Johnson A.E.W., Schwendicke F., Quinonez C. (2024). Detecting dental caries on oral photographs using artificial intelligence: A systematic review. Oral Dis..

[28] Li R., Zhu J., Wang Y., Zhao S., Peng C., Zhou Q., Sun R., Hao A., Li S., Wang Y. (2021). Development of a deep learning based prototype artificial intelligence system for the detection of dental caries in children. Chin. J. Stomatol..

[29] Bayraktar Y., Ayan E. (2022). Diagnosis of interproximal caries lesions with deep convolutional neural network in digital bitewing radiographs. Clin. Oral Investig..

[30] Schwendicke F., de Oro J.C.G., Garcia Cantu A., Meyer-Lueckel H., Chaurasia A., Krois J. (2022). Artificial intelligence for caries detection: Value of data and information. J. Dent. Res..

[31] Bader J.D., Shugars D.A. (2006). The Evidence Supporting Alternative Management Strategies For Early Occlusal Caries and Suspected Occlusal Dentinal Caries. J. Evid. Based Dent. Pract..

[32] Aps J., Lim L., Tong H., Kalia B., Chou A. (2020). Diagnostic efficacy of and indications for intraoral radiographs in pediatric dentistry: A systematic review. Eur. Arch. Paediatr. Dent..

[33] Caceda J.H., Jiang S., Calderon V., Villavicencio-Caparo E. (2023). Sensitivity and specificity of the ICDAS II system and bitewing radiographs for detecting occlusal caries using the Spectra^TM^ caries detection system as the reference test in children. BMC Oral Health.

[34] Bayati M., Alizadeh Savareh B., Ahmadinejad H., Mosavat F. (2025). Advanced AI-driven detection of interproximal caries in bitewing radiographs using YOLOv8. Sci. Rep..

[35] Ulya S., Santoso H.A., Basuki R.S., Syifa L.L. A Comparative Study of YOLOv7 and YOLOv8 for Identification of Dental Caries on Intraoral RGB Images. Proceedings of the 2025 International Seminar on Application for Technology of Information and Communication (iSemantic).

[36] Kamburoglu K., Kolsuz E., Murat S., Yüksel S., Ozen T. (2012). Proximal caries detection accuracy using intraoral bitewing radiography, extraoral bitewing radiography and panoramic radiography. Dentomaxillofacial Radiol..

[37] Jin L., Zhou W., Tang Y., Yu Z., Fan J., Wang L., Liu C., Gu Y., Zhang P. (2024). Detection of C-shaped mandibular second molars on panoramic radiographs using deep convolutional neural networks. Clin. Oral Investig..

[38] Çelebi A., Küçük D.B., Adaş G.N., Küçük A.Ö., Türkoğlu M., Atıl F. (2025). Panoramik Radyografilerde Foramen Mentalenin Yapay Zeka Tabanlı Sistemler ile Tespiti. J. Health Inst. Türkiye.

[39] Yiğit M.K., Akyol R., Yalvaç B., Dilek F., Canger E.M. (2023). How Available are Panoramic Radiographs in the Diagnosis of Interproximal Caries? A Study with Dental Students and Dentists. Türk Diş Hekim. Araştırma Derg..

[40] Güneç H.G., Ürkmez E.Ş., Danaci A., Dilmaç E., Onay H.H., Aydin K.C. (2023). Comparison of artificial intelligence vs. junior dentists’ diagnostic performance based on caries and periapical infection detection on panoramic images. Quant. Imaging Med. Surg..

[41] Tichý A., Kunt L., Nagyová V., Kybic J. (2024). Automatic caries detection in bitewing radiographs-Part II: Experimental comparison. Clin. Oral Investig..

