# Supplementary material for: Deep Learning-Based Dental Caries Diagnosis on Panoramic Radiographies: Performance of YOLOv8 Versus Human Observers
Source: Diagnostics (Basel). 2026 Apr 13;16(8):1150. doi: 10.3390/diagnostics16081150 (PMC13114539; doi:10.3390/diagnostics16081150)
Supplement: Supplementary file 1 [file diagnostics-16-01150-s001.zip › diagnostics-4168753-supplementary.pdf]

Deep Learning–Based Dental Caries Diagnosis on Panoramic Radiographies: Performance of YOLOv8 Versus Human Observers

Supplementary information tables

Table S1. Pairwise comparison of prediction disagreement between the AI model and human observers using the exact McNemar test

**Lesion type**	**Comparison**	***n***	**Discordant predictions (AI yes / comparator yes)**	**Exact McNemar p**	**BH-adjusted p**
Buccal caries	AI vs NSS	800	0 / 61	<0.001	<0.001
Buccal caries	AI vs ESS	800	0 / 19	<0.001	<0.001
Buccal caries	AI vs ID	800	0 / 136	<0.001	<0.001
Approximal caries	AI vs NSS	800	80 / 59	0.089	0.098
Approximal caries	AI vs ESS	800	90 / 36	<0.001	<0.001
Approximal caries	AI vs ID	800	105 / 37	<0.001	<0.001
Occlusal caries	AI vs NSS	800	13 / 114	<0.001	<0.001
Occlusal caries	AI vs ESS	800	16 / 53	<0.001	<0.001
Occlusal caries	AI vs ID	800	19 / 128	<0.001	<0.001
Overall pooled	AI vs NSS	2400	93 / 234	<0.001	<0.001
Overall pooled	AI vs ESS	2400	106 / 108	0.946	0.946
Overall pooled	AI vs ID	2400	124 / 301	<0.001	<0.001

Discordant predictions indicate cases in which the AI model and the comparator gave different binary decisions, irrespective of reference correctness. ID: Intern Dentist; NSS: Novice Specialist Student; ESS: Experienced Specialist Student; AI: Artificial intelligence.
